# A survey on data augmentation for EEG-based emotion recognition and cognitive workload decoding

**DOI:** 10.3389/fnins.2026.1789468

**Published:** 2026-04-10

**Authors:** Yunyu Zhu, Yueying Zhou, Pengpai Wang, Lishan Qiao

**Affiliations:** 1 School of Mathematics and Systems Science, Liaocheng University, Liaocheng, China; 2 School of Computer and Information Engineering, Nanjing Tech University, Nanjing, China; 3 School of Computer Science and Technology, Shandong Jianzhu University, Jinan, China

**Keywords:** cognitive workload, data augmentation, deep learning, electroencephalography (EEG), emotion

## Abstract

Electroencephalography (EEG) is extensively employed in emotion recognition and cognitive workload decoding. However, signal characteristics and inter-subject variability pose significant challenges for deep learning models, particularly due to data scarcity and limited generalization. Although data augmentation (DA) is a critical approach to addressing data scarcity, a notable paucity of systematic reviews exists within deep learning frameworks focused exclusively on these two tasks. Through a systematic review of relevant literature, we summarize commonly used public EEG datasets, input representations, and deep learning classifiers. Subsequently, we focus on analyzing the specific applications and effectiveness of seven categories of DA methods in emotion recognition and cognitive workload decoding. The investigation identifies current challenges in this field, explores future research directions, and provides valuable references for researchers seeking to select and apply DA techniques to enhance model performance.

## 1 Introduction

Electroencephalography (EEG) is widely employed in emotion recognition and cognitive workload decoding due to its high temporal resolution, non-invasiveness, and capacity to directly reflect neural activity (Alarcao and Fonseca, 2019; Zhou et al., 2022; Antonenko et al., 2010). Emotion recognition plays a pivotal role in domains such as mental health monitoring (Uyanik et al., 2025; Liu et al., 2022b; Schaefer et al., 2010) and neurofeedback therapy (Huang et al., 2023; Miranda-Correa et al., 2021). Cognitive workload decoding finds extensive application in driver fatigue warning systems (Zhou et al., 2025c; Zyma et al., 2019), brain-computer interface optimization (Karmakar et al., 2025; Lim et al., 2018), and other safety-critical scenarios (Zhou et al., 2025a). However, EEG signals exhibit inherent characteristics including non-stationarity, low signal-to-noise ratio, and high dimensionality. These properties pose significant challenges for EEG-based deep learning models, notably concerning data scarcity and poor cross-subject generalization capability (Wang et al., 2025a; Jiang et al., 2025).

Deep learning, as an important branch in the field of artificial intelligence, has made rapid development in recent years. It realizes automatic extraction of complex features and pattern recognition by simulating the neural network structure of the human brain (LeCun et al., 2015). With its superior capabilities, deep learning models have made many advancements in the field of EEG analysis. In EEG-based emotion recognition, deep learning models have effectively improved classification performance through spiking neural networks that integrate adaptive graph convolution and long short-term memory (LSTM) to capture spatial–temporal dynamics (Gong et al., 2023). For cognitive workload decoding across time and subjects, a bi-classifier joint domain adaptation model is used to align domain distributions while preserving category discrepancies, significantly improving cross-domain generalization (Shao et al., 2024).

Conventional deep learning models typically require large-scale training data (Guo et al., 2025; He et al., 2021). The high acquisition costs of EEG data, complex experimental paradigms (e.g., eliciting specific emotional or cognitive states), and reliance on multimodal data (e.g., subjective reports, facial expressions, or physiological signals) for expert annotations collectively restrict the scale of available datasets (Choo et al., 2024; Alakus et al., 2020; Bird et al., 2018). Furthermore, inter-subject variability in EEG characteristics compounds model generalization challenges (Li W. et al., 2025). Consequently, developing effective EEG data augmentation (DA) methods to expand and enrich the diversity of training samples has become a critical pathway for enhancing the robustness and generalization capability of EEG-based deep learning models.

DA expands datasets by generating new samples through transformations of existing data, thereby enhancing model generalization capability and robustness (Lashgari et al., 2020; Yang et al., 2023). In fields such as computer vision and the Internet of Things, common DA approaches include applying geometric transformations to the training data and injecting random noise. For instance, Yang et al. (2023) enhanced data diversity in tasks like image classification and object detection by applying geometric transformations (e.g., translation, rotation, cropping, scaling) and injecting random perturbations into raw image data. In radar-based human activity recognition, researchers further enriched the training data through DA methods such as random cropping, horizontal flipping, and the introduction of Gaussian noise, thereby enhancing the model’s generalization ability and robustness to environmental variations (Li et al., 2024).

Recent years have witnessed systematic reviews of DA methods for diverse EEG applications, including motor imagery (MI) and disease diagnosis. For example, Keutayeva and Abibullaev (2024) primarily focused on MI decoding, reviewing three categories of DA techniques used in transformer-based EEG classification, including geometric transformations, generative models, and feature transformations. Similarly, Lashgari et al. (2020) surveyed EEG DA methods in deep learning architectures, yet their literature predominantly covered epileptic seizure detection and MI tasks. They classified the DA methods used across various tasks into seven categories: noise addition, generative adversarial networks (GANs), sliding window, sampling, Fourier transform, recombination of segmentation, and other.

Although several review studies have discussed DA techniques in EEG and related biomedical signal analysis, surveys specifically focusing on emotion recognition and cognitive workload decoding within deep learning frameworks remain limited. Most existing reviews have focused on broader application domains, such as MI or general EEG classification (Lashgari et al., 2020; Rommel et al., 2022), rather than these two tasks. Furthermore, they mainly provided descriptive summaries of augmentation methods, with relatively limited discussion of how augmentation effectiveness may vary across EEG input representations and evaluation settings (Hassan et al., 2025). Consequently, the practical applicability and methodological validity of DA strategies for these tasks remain insufficiently clarified.

Moreover, although DA is widely regarded as a promising strategy for alleviating data scarcity and improving model robustness, its application to EEG is not straightforward. Unlike natural images, EEG signals contain physiologically meaningful temporal, spectral, and spatial relationships (Zhong et al., 2022). Inappropriate augmentation may distort such relationships, alter topology-related information, or generate samples that are not physiologically plausible (Lashgari et al., 2020; Zhong et al., 2022). In addition, operations such as overlapping segmentation, within-trial sample expansion, or excessive perturbation may introduce artificial correlations between samples and weaken the assumption of sample independence (Brookshire et al., 2024; Del Pup et al., 2025). These issues are particularly important when evaluating DA under subject-dependent settings, where apparent performance gains may partly reflect optimistic data partitioning rather than true improvements in cross-subject generalization (Brookshire et al., 2024; Del Pup et al., 2025; Apicella et al., 2024). Therefore, a review of EEG DA should not only summarize reported performance improvements, but also critically examine the methodological assumptions, risks, and generalizability of different augmentation strategies.

In this context, the present review focuses specifically on DA methods for deep learning-based EEG emotion recognition and cognitive workload decoding. Rather than providing a general overview of all EEG augmentation studies, this survey is restricted to strategies designed to expand training data and enhance model learning in these two decoding tasks. Building on this task-specific focus, we systematically organize existing DA techniques, analyze their comparative advantages and limitations under different EEG input representations and evaluation settings, and discuss methodological challenges as well as future research directions. The primary contributions are fourfold:

1) To provide a focused review of DA methods for EEG-based emotion recognition and cognitive workload decoding.
2) To provide a structured analysis of the literature by datasets, EEG input representations, deep learning classifiers, and evaluation settings together.
3) To compare the practical utility and limitations of different DA strategies across representation types and validation protocols.
4) To highlight methodological challenges and suggest future directions for more reliable and generalizable EEG decoding research.

The structural organization of this review proceeds as follows: section 1 examines the research background and motivation. Section 2 delineates the literature retrieval strategy and inclusion criteria. Section 3 comprehensively describes four open-access EEG datasets, followed by a synthesis of deep learning classification architectures and their input representations for emotion recognition and cognitive workload decoding. Additionally, this section introduces the fundamental principles of EEG DA methods and their practical implementations as found in the existing literature. Section 4 critically examines prevailing challenges and proposes future research avenues. Conclusively, section 5 provides the conclusions of this paper.

## 2 Methods

### 2.1 Search method

**Figure 1:**
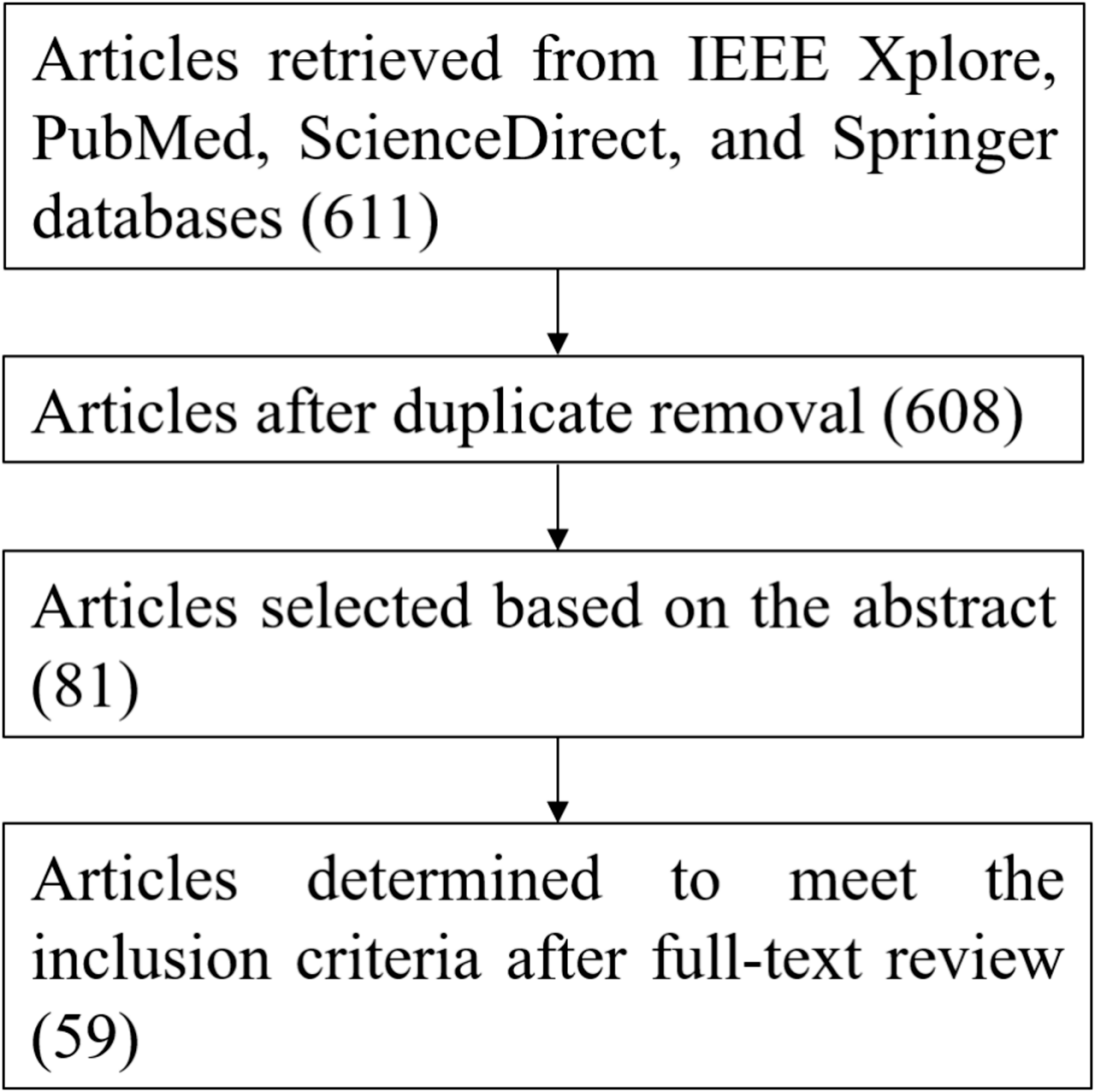
The screening process of articles.

The search was conducted on 25 May 2025 within the IEEE Xplore, PubMed, ScienceDirect, and Springer databases using the following group of keywords: (“Data Augmentation”) AND (“Deep Neural Network” OR “Deep Learning” OR “Deep Machine Learning” OR “Deep Convolutional” OR “Representation Learning” OR “Deep Recurrent” OR “Deep LSTM”) AND (“EEG” OR “Electroencephalography”) AND (“Emotion” OR “Workload”). Table 1 lists the inclusion and exclusion criteria. The literature screening workflow is depicted in Figure 1. As shown, after removal of cross-database duplicates and publications meeting exclusion criteria, 59 articles satisfying inclusion criteria were retained for analysis.

**Table 1:** Inclusion and exclusion criteria.

Inclusion criteria	Exclusion criteria
Publications within the last 5 years Studies on emotion recognition or cognitive workload tasks Non-invasive EEG signal analysis Deep learning-based decoding architectures DA techniques for sample expansion	Review articles Research for invasive EEG, electrocardiogram (ECG), functional magnetic resonance imaging, and so on, or multimodal biosignal studies Research not involving classification

### 2.2 Data extraction and presentation

For all datasets cited in the literature, we extracted the following features: types of tasks, types of stimuli, number of electrodes, sampling rate, number of subjects, number of trials, trial duration, and ratings/levels. The dataset information is detailed in Table 2.

**Table 2:** Details of dataset information.

Task	Dataset/Source	Stimuli	#*N*	Sampling rate	#*S* (M/F)	#*T*	#D	Ratings/Levels
Emotion recognition	DEAP (Koelstra et al., 2012)	Music videos	32	512 Hz	32 (16/16)	40	60 s	Valence (1–9), arousal (1–9), dominance (1–9), liking (1–9), familiarity (1–5)
	SEED (Zheng and Lu, 2015; Duan et al., 2013)	Movie clips	62	1,000 Hz	15 (7/8)	45	4 m	Negative, positive, neutral
	SEED-IV (Zheng et al., 2019)	Movie clips	62	1,000 Hz	15 (7/8)	72	2 m	Happy, sad, neutral, fear
	SEED-V (Liu et al., 2022a)	Movie clips	62	1,000 Hz	20 (10/10)	45	2–4 m	Happy, sad, disgust, neutral, fear
	SEED-VII (Jiang et al., 2025)	Movie clips	62	1,000 Hz	20 (10/10)	80	2–5 m	Happy, sad, neutral, disgust, fear, surprise, anger
	SEED-FRA (Liu et al., 2022b; Schaefer et al., 2010)	Movie clips	62	1,000 Hz	8 (5/3)	63	1–4 m	Negative, positive, neutral
	SEED-GER (Liu et al., 2022b; Schaefer et al., 2010)	Movie clips	62	1,000 Hz	8 (7/1)	63	1-4 m	Negative, positive, neutral
	DREAMER (Katsigiannis and Ramzan, 2018)	Movie clips	14	128 Hz	23 (14/9)	18	65–393 s	Valence (1–5), arousal (1–5), dominance (1–5)
	MAHNOB-HCI (Soleymani et al., 2012)	Videos	32	256 Hz	27 (11/16)	20	34.9–117 s	Valence (1–9), arousal (1–9), dominance (1–9)
	AMIGOS (Miranda-Correa et al., 2021)	Long videos	14	128 Hz	37	16	250 s (max)	Valence (1–9), arousal (1–9), dominance (1–9), liking (1–9), familiarity (1–9) and neutral, disgust, happiness, surprise, anger, fear, sadness
		Short videos	14	128 Hz	40	4	14 m (min)	
	GAMEEMO (Alakus et al., 2020)	Computer games	14	128 Hz	28	4	5 m	Boring, calm, horror, funny
	EEG-brainwave (Bird et al., 2018)	Music and the shell game	4	150-270 Hz	4 (2/2)	3	1 m	Relaxing, neutral, concentrating
	Kim and Im (2024)	Videos	32	2048 Hz	68 (38/30)	8	120 s	Happiness (1–5), sadness (1–5), anger (1–5), peacefulness (1–5)
	Er et al. (2021)	Music	12	128 Hz	9 (5/4)	16	30 s	Happy, sad, relax, angry
	CEED (Deng et al., 2024)	Videos	64	1,000 Hz	50 (40/10)	15	3 m	Negative, positive, neutral
Cognitive workload	STEW (Lim et al., 2018)	SIMKAP task	14	128 Hz	48 (48/0)	2	3 m	Mental workload scale (1–9)
	MV (Yin et al., 2024)	aCAMS task	11	500 Hz	8 (8/0)	12	15 m	Mental workload level (1–5)
	MAT (Zyma et al., 2019)	Arithmetic task (serial subtractions)	23	500 Hz	36 (27/9)	2	60 s/180 s	Mental workload level (low/high)
	Wang et al. (2024a)	Passive auditory stimulation task	32	1,000 Hz	10 (5/5)	8	10 m	Mental workload level (low/high)
	Jin et al. (2024)	MATB-II	32	1,024 Hz	10 (8/2)	18	12 m	Mental workload level (low/high)
	Gupta et al. (2021)	Visual shape task Visual color task	32	500 Hz	44	40	14 s (max)	Mental workload level (easy/difficult)
	Yu and Chen (2024)	Movie clips	14	128 Hz	24	3	10 m	Mental workload level (low/moderate/high)

#*N*, the number of electrodes; #*S*, the number of subjects, M, male, F, female; #*T*, the number of trials (per subject); #D, trial duration.

## 3 Techniques of cognitive state decoding

This section systematically analyzes DA techniques for EEG-based emotion recognition and cognitive workload decoding within deep learning frameworks. It begins with an analysis and introduction of the datasets, followed by a discussion of input modeling methods used to represent EEG data, as well as deep learning classification models. Finally, it offers a comprehensive classification and evaluation of the DA methods.

### 3.1 Dataset

**Figure 2:**
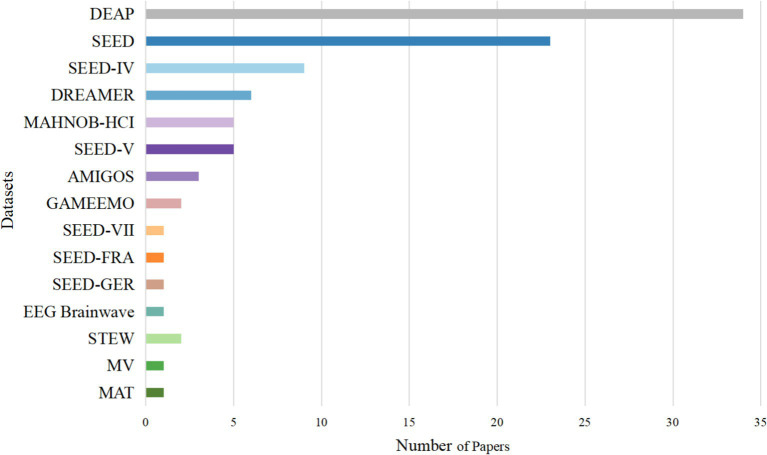
Number of public datasets across the reviewed studies.

Among the 59 collected studies, 54 focused on emotion recognition and 5 addressed cognitive workload. The emotion recognition studies utilized 15 different datasets, of which 12 were publicly available, and 3 were private. The cognitive workload studies employed 3 publicly available datasets and 4 private datasets. Figure 2 illustrates the number of publicly available datasets utilized in the reviewed studies.

Given the large number of datasets identified in the literature, it is impractical to describe all of them in detail. Therefore, this review focuses on representative public datasets that are most frequently used in the collected studies. Although datasets from both emotion recognition and cognitive workload studies were considered in the statistics, the four most frequently used datasets are all related to emotion recognition. Accordingly, these four datasets are described in detail below.

DEAP (Koelstra et al., 2012): Developed by Queen Mary University of London, this dataset comprises EEG signals from 32 electrodes and peripheral physiological signals from 8 channels. Thirty-two participants (16 males, 16 females; aged 19–37 years) were recruited. Each participant viewed 40 one-minute music video clips to elicit emotional responses, while physiological data were recorded using a Biosemi ActiveTwo system at 512 Hz. After the stimulus, participants performed self-assessments using the self-assessment manikin (SAM) across five dimensions: valence, arousal, dominance, and liking (9-point scale, 1–9), and familiarity (5-point scale, 1–5). Frontal facial videos were recorded for the first 22 participants only.

SEED series: The SEED (Zheng and Lu, 2015; Duan et al., 2013), SEED-IV (Zheng et al., 2019), and SEED-V (Liu et al., 2022a) datasets were provided by the Brain and Cognitive Science Laboratory of Shanghai Jiao Tong University. All three datasets included 62 channels of EEG signals and eye movement data, with the EEG sampled at 1,000 Hz.

1) SEED: Fifteen movie clips with three emotions (neutral, positive, and negative) were selected as stimuli, with each emotion corresponding to 5 movie clips. Fifteen subjects (7 males, 8 females) were exposed to three separate experiments, each consisting of 15 movie clips. Individual clips lasted approximately 4 min. Each clip was preceded by a 5 s cue and followed by a 45 s self-assessment and a 15 s break.
2) SEED-IV: This dataset used 72 movie clips with four emotions (sad, fearful, happy, and neutral) as stimuli. The experiment was completed by 15 subjects (7 males and 8 females). Each subject performed three experiments with completely different stimuli. Each experiment consisted of 24 movie clips (6 clips per emotion). Movie clips were approximately 2 min long, each preceded by a 5 s start cue and followed by a 45 s self-assessment.
3) SEED-V: Forty-five movie clips with five emotions (happy, fearful, neutral, sad, and disgusted) were selected as stimuli. Twenty subjects (10 males, 10 females) participated in three experiments with completely different stimuli each time. Each experiment consisted of 15 movie clips (3 clips per emotion). Clip durations ranged from 2 min to 4 min. Each clip was preceded by a 15 s start cue, followed by a 15 s or 30 s self-assessment and rest period (duration material-dependent).

DREAMER (Katsigiannis and Ramzan, 2018): The dataset comprises EEG signals sampled at 128 Hz with 14 channels and ECG signals sampled at 256 Hz. Twenty-three subjects (14 males, 9 females) viewed 18 film clips, with durations between 65 s and 393 s, designed to elicit nine emotions (anger, fear, sadness, disgust, calmness, surprise, amusement, happiness, and excitement). Post-viewing, participants assessed valence, arousal, and dominance via the SAM, employing a 5-point scale.

MAHNOB-HCI (Soleymani et al., 2012): The dataset includes EEG signals sampled at 256 Hz and other physiological signals from 30 subjects (13 males, 17 females). EEG data were collected using a Biosemi ActiveTwo system with 32 channels. Due to data loss, complete records were obtained for 27 subjects. Each subject viewed 20 video clips lasting between 34.9 s and 117 s. Post-stimulus, participants rated valence, arousal, dominance, and predictability using the SAM scale (1–9).

### 3.2 Input formulation

Among the literature meeting inclusion criteria, EEG input formulations for deep learning models are primarily categorized into four types: raw signal values, extracted features, spectral images, and topological maps. Details for each study are summarized in Table 3. In end-to-end learning paradigms, raw EEG signals in the time domain serve as direct inputs to deep neural networks (DNNs). For input formulations based on extracted features, EEG classification generally follows a two-stage process: first, traditional feature extraction methods transform EEG signals into feature vectors, which are subsequently fed into deep learning models for classification. Conversion to spectral images is typically achieved via techniques such as short-time Fourier transform (STFT) and wavelet transform. These methods encode spectral information of raw signals into image representations, enabling deep learning models to process EEG data akin to image data. In topological maps, spatial information of EEG signals is structured as graph-based representations, facilitating the modeling of dynamic interactions between brain regions.

**Table 3:** Details for each study.

Study	Input formulation	Classifier	Sliding window	Other DA methods	Dataset
Griffith and Hubbard (2021)	SI	CNN	Non-overlapping	NA	DEAP
Jha et al. (2024)	EF	CNN	Non-overlapping	NA	DEAP, EEG brainwave
Kim and Im (2024)	RS	CNN	Non-overlapping	NA	Kim and Im (2024)
Jana et al. (2022)	TM	CapsNet	Non-overlapping	NA	DEAP
Wu et al. (2022)	EF	LSTM	Overlapping	NA	SEED, MAHNOB-HCI
Wei et al. (2020)	EF	SRU	Overlapping	NA	SEED
Zhang X. et al. (2024)	EF	CNN + LSTM	Overlapping	NA	SEED
Deng et al. (2024)	EF	MLP	Non-overlapping	NA	SEED, CEED
Quan et al. (2023)	RS	CNN	Non-overlapping	NA	DEAP, SEED, SEED-IV, DREAMER
Huang et al. (2021)	TM	CNN	Non-overlapping	NA	DEAP
Kuang et al. (2023)	RS	CNN	Non-overlapping	NA	DEAP, SEED
Cui et al. (2023)	TM	CNN	Non-overlapping	NA	DEAP
Wang et al. (2024b)	RS	CNN + Transformer	Non-overlapping	NA	DEAP, AMIGOS
Fan et al. (2024)	RS	CapsNet + LSTM	Non-overlapping	NA	DEAP, DREAMER
Deng et al. (2021)	RS	CapsNet + LSTM	Non-overlapping	NA	DEAP
Li J. et al. (2023)	EF	CNN	Non-overlapping	NA	SEED
Yu and Chen (2024)	RS	CNN + Transformer	Non-overlapping	NA	STEW, (Yu and Chen, 2024)
Lin et al. (2023)	EF	CNN + GNN	Non-overlapping	NA	DEAP, SEED
Li et al. (2022)	RS	CNN + LSTM	Non-overlapping	NA	AMIGOS
Li et al. (2023b)	RS	MLP + Transformer	Non-overlapping	NA	DEAP, DREAMER
Aslan et al. (2023)	EF	CNN	Overlapping	NA	DEAP, SEED-V, GAMEEMO
Bhosale et al. (2022)	TM	CNN + LSTM + ProtoNet	Overlapping	NA	DEAP
Jiang et al. (2023)	RS	CNN	Overlapping	NA	DEAP, SEED
Ahmed and Longo (2022)	TM	MLP	Overlapping	NA	DEAP
Peng et al. (2024)	TM	Transformer	Non-overlapping	NA	DEAP
Moreno-Alcayde et al. (2024)	RS	CNN	Both types	NA	DEAP
Szczakowska et al. (2023)	RS	CNN	Both types	Noise addition	MAHNOB-HCI
Gupta et al. (2021)	EF	LSTM	Overlapping	Noise addition	Gupta et al. (2021)
Zhang et al. (2022)	EF	CNN	Non-overlapping	Noise addition	SEED, SEED-IV, SEED-V, AMIGOS
Moghadam and Hegazy (2025)	EF	CNN	NA	Noise addition, pair averaging, multi-sample averaging	SEED-VII
Yin et al. (2024)	TM	CNN	Non-overlapping	Noise addition, flipping	STEW, MV, MAT
Almanza-Conejo et al. (2023)	SI	CNN	NA	Noise addition	DEAP
Kamble and Sengupta (2024)	SI	CNN	Overlapping	Noise addition, scaling, brightness adjustment	DREAMER, SEED, SEED-IV
Cai et al. (2024)	SI	Transformer	Non-overlapping	Flipping, rotation, resized crop	SEED, SEED-IV
Khan et al. (2024)	SI	CNN	NA	Flipping, rotation	SEED
Er et al. (2021)	SI	CNN	Overlapping	Noise addition, color shifting, rotation, shearing	Er et al. (2021)
Mane and Shinde (2023)	TM	CNN + LSTM	NA	Noise addition, flipping, translation, scaling	DEAP, SEED
Gilakjani and Osman (2024)	EF	GNN	Non-overlapping	GAN	DEAP, MAHNOB-HCI
Qiao et al. (2024)	TM	CNN + RNN	Non-overlapping	GAN	SEED-IV, DREAMER
Tian et al. (2023)	EF, TM	MLP	Overlapping	VAE + GAN	SEED
Zhang et al. (2023)	TM	CNN	Non-overlapping	GAN	DEAP, SEED, DREAMER
Ari et al. (2022)	SI	CNN	NA	AE	GAMEEMO
Kalashami et al. (2022)	EF	MLP	NA	GAN	DEAP
Pan and Zheng (2021)	TM	CNN	Overlapping	GAN	DEAP, MAHNOB-HCI
Luo et al. (2020)	EF	MLP	Non-overlapping	VAE + GAN	DEAP, SEED
Zhu et al. (2024)	TM	CNN + Transformer	Non-overlapping	VAE + GAN	DEAP, SEED
Wan et al. (2025)	TM	CNN + Transformer	Non-overlapping	Generative transformer	DEAP, SEED, SEED-IV
Chen et al. (2021)	EF	CNN	Overlapping	Borderline-SMOTE	DEAP
Singh K. et al. (2023)	RS	CNN + LSTM	Non-overlapping	SMOTE	DEAP
Singh U. et al. (2023)	EF	LSTM	Non-overlapping	EMD	DEAP
Aldawsari et al. (2023)	EF	CNN	Non-overlapping	Graph EMD	DEAP, SEED, MAHNOB-HCI
Wang et al. (2024a)	RS	CNN + LSTM	Overlapping	FTS, Mixup	Wang et al. (2024a)
Zhou R. et al. (2025)	EF	MLP	Non-overlapping	Mixup	SEED, SEED-IV, SEED-V
Jin et al. (2024)	EF	CNN	Non-overlapping	Random temporal disruption	Jin et al. (2024)
Bagherzadeh et al. (2024)	SI	CNN	Non-overlapping	Cross-subject channel sampling	SEED-IV, SEED-V, SEED-FRA, SEED-GER
Kan et al. (2022)	RS	CNN	Non-overlapping	Meiosis	DEAP, SEED
Kang et al. (2022)	SI	CNN + RNN	Overlapping	ICA-E	DEAP
Zhang D. et al. (2024)	SI	CNN	NA	Multi-scale wavelet decomposition and recombination	DEAP
Chen et al. (2025)	TM	GNN	NA	Uncertainty-guided statistical transformation	SEED, SEED-IV, SEED-V

For input formulation, RS, raw signal; EF, extracted features; SI, spectral images; TM, topological maps. For other DA methods, NA, not applicable.

### 3.3 Classification model based on deep learning

With the rapid advancement of deep learning, its models have been extensively applied in EEG signal research, providing powerful analytical tools for brain-computer interfaces (Liu X.-Y. et al., 2025), cognitive state monitoring (Zhou et al., 2025b), and neurological disease diagnosis (Uyanik et al., 2025). Through systematizing the collected literature, we categorize deep learning models applied to EEG-based emotion recognition and cognitive workload decoding into six classes: convolutional neural networks (CNNs), recurrent neural networks (RNNs), multilayer perceptrons (MLPs), graph neural networks (GNNs), transformers, and hybrid models.

CNNs, leveraging exceptional local feature extraction capabilities, are the most widely applied models in EEG classification. Their canonical structure comprises convolutional layers, pooling layers, and fully connected layers (Paliwal et al., 2024). Convolutional layers employ kernels to scan input data, effectively capturing local spatiotemporal features. Pooling layers perform downsampling to reduce dimensionality while preserving critical information. Fully connected layers map extracted features for final classification. To better fit the spatio-temporal properties of EEG signals, researchers have proposed various improved CNN architectures. For example, Griffith and Hubbard (2021) adopted a deep residual network architecture, which introduces residual connections into the CNN framework to construct deeper convolutional networks, ultimately enabling classification of feature maps. Jha et al. (2024) employed a multi-column CNN with a multi-branch structure to process EEG data from different channels in parallel. Kim and Im (2024) proposed a novel CNN architecture named the Hilbert-transform and raw EEG network. Capsule neural network (CapsNet) improves traditional CNNs’ feature extraction capability by introducing capsules and a dynamic routing mechanism, better preserving spatial hierarchies and enhancing model robustness (Jana et al., 2022).

RNNs, characterized by their unique recurrent structure, excel at capturing temporal dependencies and dynamic patterns within EEG signals. The core mechanism of RNNs lies in the recursive transmission of hidden states, which integrates past information into the computation of the current timestep, thereby modeling dependencies in time series data (Sreedhar et al., 2025). However, traditional RNNs often encounter difficulties such as vanishing or exploding gradients when processing long sequences, hindering their ability to learn long-term dependencies (Majeed et al., 2025). To address this limitation, LSTMs and gated recurrent units (GRUs) incorporate gating mechanisms that effectively regulate information flow and memory updates. Wu et al. (2022) processed EEG signals through independent component analysis (ICA) and Riemannian manifold methods, then employed a two-layer LSTM for emotion classification. Wei et al. (2020) adopted a two-layer simple recurrent unit (SRU) architecture, enhancing the capability of EEG emotion recognition through an efficient RNN variant.

MLPs represent the most fundamental DNN architecture, composed of multiple fully connected layers (Tolstikhin et al., 2021). Each layer transforms inputs through nonlinear activation functions, enabling the learning and nonlinear mapping of features. For instance, Li et al. (2023a) proposed a hierarchical three-dimensional MLP-based model, which reconstructs EEG signals into period-channel-time three-dimensional tensors and uses stacked three-dimensional MLP blocks to hierarchically extract cross-period spatiotemporal features, enhancing cross-subject emotion recognition performance. In recent years, transformer models have demonstrated remarkable potential by leveraging their powerful self-attention mechanism. Their core strength lies in the ability to capture global dependencies between any positions within a sequence through this self-attention mechanism. Zheng and Pan (2024) proposed a spatiotemporal symmetric transformer model, which automatically extracts spatiotemporal features for emotion recognition. Liu T. et al. (2025) designed a dual-branch network to classify drivers’ mental workload levels. This network captures topological relationships among EEG channels through its graph attention branch and extracts temporal-spectral features via a multi-head self-attention branch, achieving excellent classification performance through feature-level fusion. GNNs, as specialized deep learning tools for processing graph-structured data, derive their principal value from the capacity to explicitly model topological connectivity relationships and functional coupling characteristics between brain electrodes (Wang H. et al., 2025). This capability provides a powerful analytical framework for gaining deeper insights into the connectivity mechanisms underlying brain activity. Gilakjani and Osman (2024) combined the topological structure of EEG channels to learn and extract relationships between EEG features through a GNN model for emotion recognition.

Hybrid deep learning models are increasingly adopted for decoding the multi-scale spatiotemporal representations within EEG signal. Their core concept centers on integrating the strengths of two or more distinct types of deep learning models, thereby overcoming the limitations inherent in single-model approaches. Zhang X. et al. (2024) proposed a method of ant colony optimization with CNN and LSTM. This method dynamically selects optimal EEG channels using ant colony optimization, constructs lightweight data, and achieves high-accuracy emotion classification.

### 3.4 DA methods

DA expands the existing training dataset by generating diverse new samples. Its primary objectives are to increase the size and diversity of the data, enhance the model’s generalization ability and robustness, and address issues such as data insufficiency, class imbalance, and overfitting (Lashgari et al., 2020; Yang et al., 2023). Among the 59 studies reviewed, deep learning-based EEG DA methods are categorized into seven main types: sliding window, noise addition, image transformation, generative modeling, sampling, empirical mode decomposition (EMD), and others.

**Figure 3:**
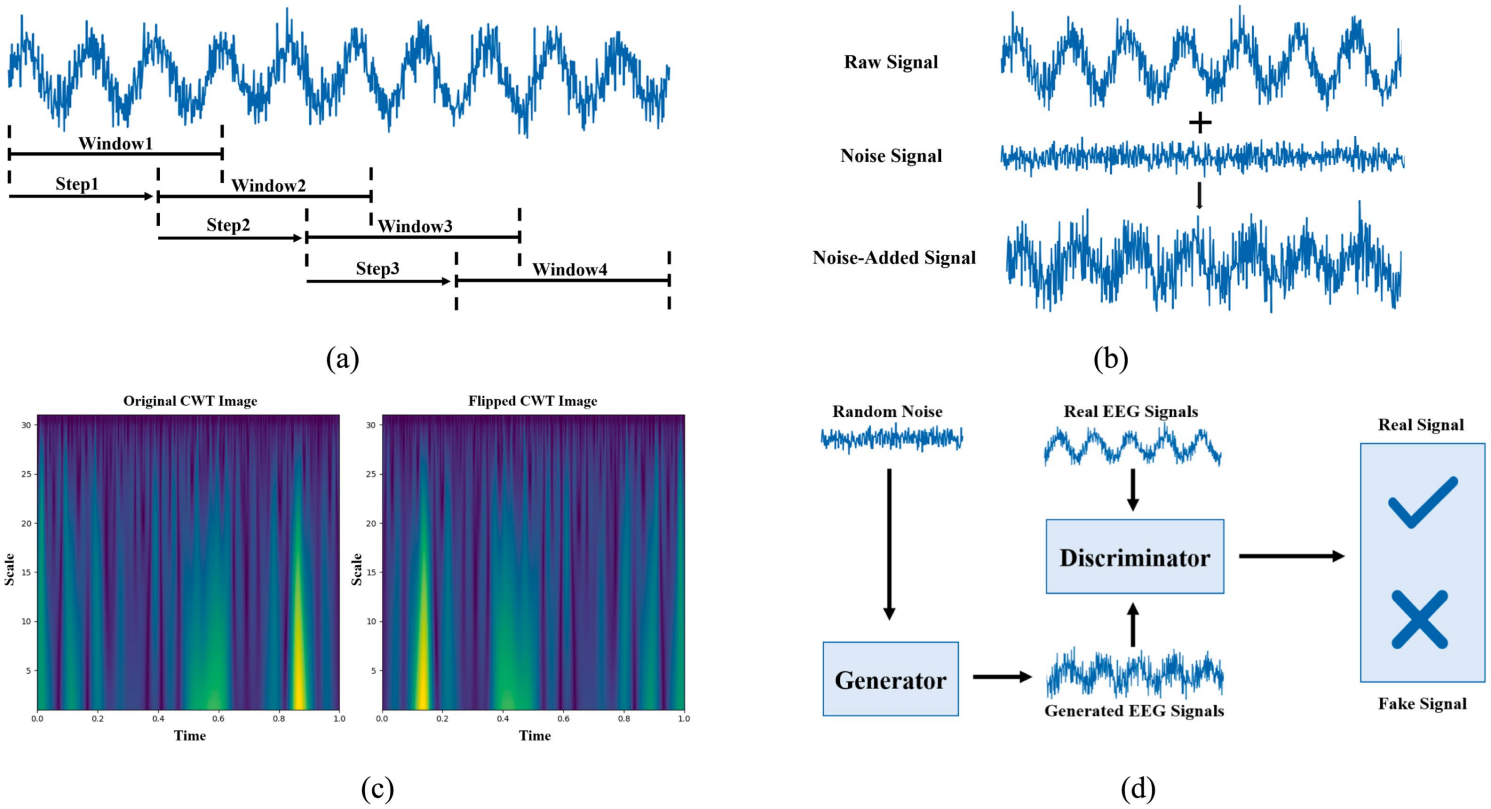
DA techniques: **(a)** sliding window, **(b)** noise addition, **(c)** image transformation, and **(d)** generative modeling.

Table 4 summarizes the main characteristics of the DA methods identified in the studies. Overall, sliding window segmentation is the most frequently adopted approach due to its simplicity and ability to rapidly expand training samples. Noise addition and image transformation are commonly used to improve model robustness. More advanced strategies, such as generative models and EMD-based methods, aim to increase data diversity by generating new samples or reconstructing signals from decomposed components. Sampling techniques mainly target class imbalance problems, but contribute limited diversity to the dataset. These observations indicate that existing EEG DA strategies vary considerably in their complexity, application level, and effectiveness, highlighting the need for careful selection based on specific tasks and model architectures. Figure 3 illustrates some of the DA techniques. The sliding window technique dominates as the most commonly used DA method in research, while sampling, EMD, and other methods remain less explored, as shown in Table 4. Below, we will introduce each of these DA techniques in detail.

**Table 4:** Comparison of EEG DA methods in the literature.

DA methods	Main purpose	Advantages	Limitations	Study
Sliding window	Increase samples	Simple, widely used	Risk of data leakage	Griffith and Hubbard (2021), Jha et al. (2024), Kim and Im (2024), Jana et al. (2022), Wu et al. (2022), Wei et al. (2020), Gilakjani and Osman (2024), Zhang X. et al. (2024), Szczakowska et al. (2023), Deng et al. (2024), Quan et al. (2023), Huang et al. (2021), Kuang et al. (2023), Cui et al. (2023), Wang et al. (2024b), Fan et al. (2024), Deng et al. (2021), Li J. et al. (2023), Yu and Chen (2024), Lin et al. (2023), Li et al. (2022), Li et al. (2023b), Aslan et al. (2023), Bhosale et al. (2022), Jiang et al. (2023), Ahmed and Longo (2022), Peng et al. (2024), Moreno-Alcayde et al. (2024), Gupta et al. (2021), Zhang et al. (2022), Yin et al. (2024), Kamble and Sengupta (2024), Cai et al. (2024), Er et al. (2021), Qiao et al. (2024), Tian et al. (2023), Zhang et al. (2023), Pan and Zheng (2021), Luo et al. (2020), Zhu et al. (2024), Wan et al. (2025), Chen et al. (2021), Singh K. et al. (2023), Singh U. et al. (2023), Aldawsari et al. (2023), Wang et al. (2024a), Zhou R. et al. (2025), Jin et al. (2024), Bagherzadeh et al. (2024), Kan et al. (2022), and Kang et al. (2022)
Noise addition	Improve robustness	Easy to implement	May distort the signal	Szczakowska et al. (2023), Gupta et al. (2021), Zhang et al. (2022), Moghadam and Hegazy (2025), Yin et al. (2024), Almanza-Conejo et al. (2023), Kamble and Sengupta (2024), Er et al. (2021), Mane and Shinde (2023)
Image transformation	Balance classes	Effective for CNN	May break the EEG topology	Yin et al. (2024), Kamble and Sengupta (2024), Cai et al. (2024), Khan et al. (2024), Er et al. (2021), and Mane and Shinde (2023)
Generative modeling	Generate new data	Increase diversity	Training complexity	Gilakjani and Osman (2024), Qiao et al. (2024), Tian et al. (2023), Zhang et al. (2023), Ari et al. (2022), Kalashami et al. (2022), Pan and Zheng (2021), Luo et al. (2020), Zhu et al. (2024), and Wan et al. (2025)
Sampling	Solve class imbalance	Improves minority classes	Limited diversity	Chen et al. (2021) and Singh K. et al. (2023)
EMD	Decompose signal	Preserve frequency info	Computational cost	Singh et al. (2023) and Aldawsari et al. (2023)
Others	Task-specific enhancement	Flexible implementations	Lack of generalization	Moghadam and Hegazy (2025), Wang et al. (2024a), Zhou R. et al. (2025), Jin et al. (2024), Bagherzadeh et al. (2024), Kan et al. (2022), Kang et al. (2022), Zhang D. et al. (2024), and Chen et al. (2025)

#### 3.4.1 Sliding window

Sliding window operates on time series data by moving a fixed-size window with a fixed step size to perform DA (Cui et al., 2016). The overlapping window is a type of sliding window. During the sliding process, when the step size is smaller than the window size, there is partial overlap between the new window and the previous one, thus forming an overlapping window (Szczakowska et al., 2023).

In data preprocessing, the sliding window technique not only helps retain most of the original information but also effectively increases both the quantity and diversity of data. According to the literature we reviewed, 86.44% of studies employed the sliding window method. Some studies, such as those by Deng et al. (2024), Quan et al. (2023), Huang et al. (2021), Kuang et al.(2023), Cui et al. (2023), Wang et al. (2024b), Fan et al. (2024), and Deng et al. (2021), used non-overlapping 1 s time windows to segment the data, while others, such as Li J. et al. (2023), Yu and Chen (2024), Lin et al. (2023), Li et al. (2022), and Li et al. (2023b), applied non-overlapping time windows of various lengths during preprocessing. Additionally, other research used overlapping windows with fixed time lengths and step sizes for DA (Aslan et al., 2023; Bhosale et al., 2022). Given the widespread application of the sliding window technique in the reviewed literature, we will conduct a detailed analysis of only a few selected studies.

Jiang et al. (2023) proposed an attention mechanism-based multiscale feature fusion network. This network first extracts spatio-temporal features from EEG signals using spatio-temporal convolution blocks, then captures emotion-related information through multiscale separable convolutions and the convolutional block attention mechanism. Experiments employed the sliding window method to increase the number of training samples. They fixed the time window at 4 s and used sliding steps of 0.5 s, 1 s, 1.5 s, 2 s, 2.5 s, and 3 s. The experiments revealed that when the sliding step was set to 1 s, the highest accuracy was achieved on the DEAP dataset. For the SEED dataset, no significant difference was observed between using 0.5 s and 1 s sliding steps.

Ahmed and Longo (2022) used a sliding window with a 125-ms step to convert EEG signals into 32 **×** 32 **×** 5 spectral topographic maps. They then constructed a convolutional variational autoencoder to learn latent spaces of varying dimensions while simultaneously training a dense neural network for classification. Experiments were conducted across different latent space dimensions (ranging from 4 to 100 in linear increments of 4) and time window lengths (0.5 s, 1 s, 1.5 s, and 2 s). The results revealed that when using topographic maps generated from 2 s time windows, a latent space of 28 dimensions achieved optimal reconstruction performance, with an average classification accuracy of 93%.

Peng et al. (2024) combined a spectrum-based spatial channel attention mechanism with a transformer incorporating a time-continuity encoding mechanism to design the EEG emotion recognition model SST-Emo. They conducted experiments on the DEAP dataset using 1 s and 3 s non-overlapping time windows. Under the 1 s window, the accuracies for arousal, valence, dominance, liking, and the 5-class task reached 96.28, 95.25, 95.52, 95.83, and 93.88%, respectively. For the 3 s window, the corresponding accuracies were 96.13, 95.74, 95.23, 95.7, and 93.83%.

Moreno-Alcayde et al. (2024) investigated the impact of different sliding window configurations on emotion recognition under three partitioning methods: subject-independent, video-independent, and time-based. They segmented EEG signals into 1 s, 2 s, or 4 s intervals and fed them into a CNN model implemented as a multi-task learning framework. Experiments classified only the valence dimension of the DEAP dataset. All three partitioning methods achieved optimal performance with non-overlapping 1 s windows, while accuracy significantly decreased when the window size increased to 4 s. They also compared different overlap ratios for 4 s windows under subject-independent conditions. With overlap ratios of 25 and 50%, performance improved notably, matching results obtained with non-overlapping 2 s or 1 s windows. However, they emphasized that overlapping windows lead to severe data leakage and should be avoided.

Overall, sliding window segmentation is the most widely used strategy in the reviewed studies due to its simplicity and its ability to quickly increase the number of training samples without altering the original signal distribution. Window lengths typically range from 1 s to 4 s, with step sizes varying across tasks and model architectures. Smaller windows capture localized temporal patterns, while moderate overlaps can increase sample density and sometimes improve classification performance (Brookshire et al., 2024; Wosiak et al., 2025). The sliding window technique implicitly assumes that EEG signals exhibit local temporal stationarity, meaning that statistical properties within a short time segment remain relatively stable (Xu et al., 2023). Under this assumption, each segmented window can be treated as an independent training sample. However, this assumption may not always hold in EEG signals due to their highly dynamic and non-stationary nature (Xu et al., 2023). In addition, overlapping windows may introduce highly correlated samples, which can lead to data leakage and overly optimistic evaluation results if not carefully separated during training and testing (Brookshire et al., 2024; Moreno-Alcayde et al., 2024).

#### 3.4.2 Noise addition

In the literature we collected, nine papers employed noise injection methods for DA, which can be categorized into three types:

a) Directly injecting interference components that simulate real noise into raw EEG signals to generate new signal samples.
b) Applying perturbations to extracted EEG features in the feature space to generate new feature vectors.
c) Injecting noise into visual representations of EEG signals (e.g., spectrograms, topographical maps). Common types of noise added include Gaussian noise and salt-and-pepper noise.

Gupta et al. (2021) proposed a cross-task cognitive workload classification framework based on temporal dynamic modeling. They added Gaussian noise at three intensities (25 dB, 30 dB, and 35 dB) to the preprocessed EEG signals and innovatively employed autoregressive modeling, fuzzy C-means clustering, and a temporal segment smoothing algorithm to construct variable-duration time frames. They then fused three types of features—band power, curve length, and approximate entropy—and input them into a DNN composed of bidirectional LSTM and LSTM for classification. Experiments demonstrated that DA enabled the model to achieve an average classification accuracy of 92.8% in cross-task testing, representing a 1.2% improvement compared to performance without DA.

Szczakowska et al. (2023) added Gaussian noise with a mean of μ ( μ = 0), standard deviation of σ ( σ = 0.05, 0.1, 0.2), and an augmentation factor of m ( m = 1, 10, 30) to preprocessed raw EEG signals. Temporal segments were constructed using sliding windows (window size = 4 s, 8 s; overlap = 0, 25, 50, 75%), which were then classified using a shallow CNN. Results demonstrated that with an 8 s sliding window, average classification accuracy for both arousal and valence dimensions was lower than with a 4 s window, and neither exceeded random classifier performance. When using overlapping windows with a 4 s window, valence accuracy increased by 4.94, 3.39, and 1.16% for 75, 50, and 25% overlap, respectively, compared to no overlap, while arousal accuracy increased by 4.8, 3.34, and 0.68%. The study confirmed that Gaussian noise with σ > 0.05 improved classification, with optimal results ( σ = 0.1, m = 30) yielding peak accuracy improvements of 8.43% for arousal and 7.72% for valence.

Zhang et al. (2022) proposed a novel semi-supervised EEG-based emotion recognition method. Their model relies on DA, label guessing, convex combinations of the unlabeled and labeled datasets, and pairwise alignment of representations between the distributions of unlabeled and labeled data. The results indicate that additive noise significantly improved model performance across all datasets. Regarding the evaluation protocol, the reported improvements on SEED, SEED-IV, and SEED-V were obtained under subject-dependent settings, whereas those on AMIGOS were obtained under a strict cross-subject protocol (leave-one-participant-out). They found that DA had the greatest impact in the nearly unsupervised scenario, with improvements of 3.7, 4.4, and 6.4% on SEED, SEED-IV, and SEED-V, respectively, and improvements of 11.2 and 9.9% on the valence and arousal dimensions of AMIGOS. However, as the amount of real unlabeled data increased, additive noise augmentation could be substituted. Therefore, although DA strategies help improve model performance, performance can be further enhanced by replacing DA with real data.

Moghadam and Hegazy (2025) engineered three EEG DA strategies for emotion recognition: pair averaging, multi-sample averaging, and Gaussian noise injection. When evaluated on the SEED-VII dataset, multi-sample averaging delivered the most significant performance gain, boosting the F1-score for seven-emotion classification by 28.6%.

Yin et al. (2024) proposed a mental workload detection framework called the shared spatial map network (SSMN). SSMN uses a shared map encoder to generate EEG feature maps with consistent spatial scalp locations. An instance augmentation encoder (IAE) then increases sample size and filters out individual-specific components through feature-space horizontal flipping and adaptive noise injection. Finally, a workload recognition committee combines shallow and deep convolutional network architectures for classification. The complete SSMN significantly outperformed variants without IAE across all validation scenarios (participant-generic, cross-participant, cross-database, and cross-session), demonstrating the effectiveness of DA.

Almanza-Conejo et al. (2023) transformed raw EEG signals into time-frequency representations (TFRs) using the continuous wavelet transform (CWT). To address the insufficient sample size for the eight-class valence-arousal-dominance (VAD) affective space, they augmented the training set by adding Gaussian white noise (parameters: μ = 0, $\sigma^{2}$ = 1) to the scalogram images, expanding the sample size to twice that of the original data. Based on a modified Google Network (GoogLeNet) architecture, they constructed classification models for both the four-class valence-arousal space and the eight-class VAD affective space. Experiments demonstrated that with DA, the validation accuracy for eight-class VAD emotion recognition reached 92.97%, an improvement of 8.71% compared to the non-augmented model.

Overall, noise addition represents one of the simplest signal-level DA techniques used in EEG analysis (Ahuja and Sethia, 2024). Noise injection methods assume that adding small perturbations does not change the underlying semantic label of EEG signals. This assumption is based on the observation that neural signals are naturally contaminated by physiological and environmental noise; therefore, moderate perturbations may help models learn more robust representations (Gordienko et al., 2025). In practice, Gaussian noise with small standard deviations is most commonly used to preserve the intrinsic statistical characteristics of EEG signals while simulating realistic signal variability and sensor noise (Szczakowska et al., 2023; Almanza-Conejo et al., 2023). Empirical studies generally report modest but consistent improvements in classification performance, particularly when training data are limited. However, excessive noise may distort important neural patterns and degrade model performance (Nair et al., 2025). Moreover, because noise-based augmentation does not introduce fundamentally new signal structures, its ability to significantly enrich data diversity is limited (Gordienko et al., 2025). Consequently, noise injection is often used in combination with other augmentation strategies to balance simplicity, robustness, and data diversity (Yin et al., 2024).

#### 3.4.3 Image transformation

In visual representations of EEG signals (e.g., time-frequency plots, topological maps), image transformation is a DA method that generates new samples by altering the geometric structure, brightness, and color of the original images (He et al., 2021). Based on the literature we collected, geometric transformations primarily include operations such as flipping, rotation, scaling, translation, and cropping, while brightness and color transformations involve adjustments to brightness levels and color shifting.

Kamble and Sengupta (2024) used the wavelet-based synchrosqueezing transform to convert one-dimensional EEG signals into two-dimensional TFRs. To address class imbalance in the dataset, they augmented the TFR images of the minority class by scaling the images within a 0.1 range, adjusting brightness levels between 0.4 and 1.5, and introducing Gaussian white noise with parameters σ = 0.2 and μ = 0, until the sample sizes matched those of the majority class. The augmented data was then input into a particle swarm optimization-weighted ensemble CNN for classification, where particle swarm optimization dynamically optimized the weight coefficients of individual CNN base classifiers. Experimental results showed that DA improved the F1-scores for the DREAMER dataset by 4.4% (arousal), 5.18% (valence), and 5.23% (dominance) compared to the non-augmented data, while area under the curve values increased by 3.15% across all dimensions. On the SEED and SEED-IV validation datasets, DA achieved accuracies of 94.67 and 89.37%, representing improvements of 0.86 and 4.8% over the non-augmented models, respectively.

Cai et al. (2024) converted EEG signals into EEG spectral images using azimuthal equidistant projection and differential entropy (DE) features. They proposed the EEG swin transformer model, which integrates a window attention mechanism and shifted window partitioning. To address cross-subject generalization, they employed DA strategies, including random horizontal flip (RHF), random vertical flip (RVF), random rotation (RR), and random resized crop (RRC). Experimental results revealed that individual DA techniques did not significantly improve accuracy on the SEED and SEED-IV datasets. Notably, RVF and RRC led to substantial declines. Subsequent combination of methods demonstrated that the RHF + RR pairing delivered optimal performance, achieving classification accuracies of 80.07% (SEED) and 66.72% (SEED-IV), representing improvements of 0.59 and 0.88% over the unaugmented baselines.

Khan et al. (2024) employed STFT and Mel-spectrograms to process EEG signals from the SEED dataset. They augmented the sample size from 675 to 10,800 using horizontal flipping and rotation. They designed an EEG-ConvNet comprising five convolutional layers and also fine-tuned the GoogLeNet and residual network-34 (ResNet-34) models. Within the EEG-ConvNet, each convolutional layer incorporated batch normalization and max pooling operations. The GoogLeNet and ResNet-34 models based on STFT achieved accuracies of 99.97 and 99.95%, respectively. The GoogLeNet and ResNet-34 models based on Mel-spectrograms attained accuracies of 99.49 and 99.31%, respectively. The proposed EEG-ConvNet achieved an accuracy of 99.03% on STFT.

Er et al. (2021) converted EEG signals into spectrograms and then employed multiple DA techniques, expanding the dataset size to six times its original scale. These techniques included random rotation, shearing, color shifting, Gaussian filtering, and salt-and-pepper noise. The augmented spectrograms were subsequently fed into pre-trained alex network (AlexNet) and visual geometry group-16 (VGG-16) networks for transfer learning. They achieved the best classification results using VGG-16 with beta band spectrograms, with an accuracy of 73.28%.

Mane and Shinde (2023) integrated a two-dimensional CNN and LSTM to propose a hybrid neural network architecture called StressNet. This model first decomposes EEG signals into α , β , and θ frequency bands. Through azimuthal projection, spectral images are generated and fed into the CNN to extract spatial features. Subsequently, temporal dynamics are captured by the LSTM. Finally, classification is performed via fully connected layers. To enhance generalization capability, they adopted bimodal DA: applying time-shifting operations and adding noise with a maximum amplitude of 20% in the signal domain, while implementing flipping, shifting, and scaling in the image domain. Ultimately, the model achieved accuracy rates exceeding 97.8% on both the DEAP and SEED datasets.

Image transformation-based DA methods are mainly applied when EEG signals are converted into visual representations such as spectrograms or topographic maps. The core theoretical assumption underlying these methods is that the discriminative information contained in EEG-derived images is approximately invariant to certain geometric or intensity transformations (Qiao and Zhao, 2025). In this context, traditional computer vision DA techniques can be directly applied to expand the dataset. Compared with signal-level augmentation, image transformations are straightforward to implement and integrate naturally with CNNs (Kamble and Sengupta, 2024; Mane and Shinde, 2023). However, excessive or inappropriate transformations may distort the physical meaning of the original signals or introduce unrealistic patterns, which can negatively affect model reliability (Zhong et al., 2022; Qiao and Zhao, 2025). Therefore, transformation operations should be carefully designed to preserve the structural characteristics of the underlying signal representations.

#### 3.4.4 Generative modeling

Generative models expand the scale and diversity of datasets by learning the distribution characteristics of data (Lei et al., 2024). Common generative models include GANs and autoencoders (AEs).

a) GAN: A GAN consists of a generator and a discriminator (Goodfellow et al., 2014). The generator synthesizes data similar to the original dataset from random inputs, while the discriminator differentiates real data from synthetic data (Kuntalp and Düzyel, 2024; Ding et al., 2024). Through adversarial training, where the generator and discriminator alternate in competition and learning, the network eventually produces a synthetic dataset that closely resembles the original data distribution (Kuntalp and Düzyel, 2024; Ding et al., 2024).
b) AE: An AE is a feedforward neural network comprising an encoder and a decoder (Li et al., 2021). The encoder compresses raw data into low-dimensional vector representations, and the decoder reconstructs these vectors to approximate the original data (Wu et al., 2024). The training process aims to minimize the error between the original inputs and the reconstructed outputs. Variants include variational autoencoders (VAEs) and denoising autoencoders (Wu et al., 2024; Huang et al., 2022).

Traditional autoencoders rely on point estimation and struggle to generate diverse samples (Wu et al., 2024). The VAE addresses this limitation by introducing probabilistic constraints, improving its generative capabilities. VAEs model the latent space as a probability distribution, typically assuming that latent variables z follow a Gaussian distribution with mean μ , variance $\sigma^{2}$ , and include a bias term ε (Kebaili et al., 2023). This approach enables VAEs to learn not only the underlying structure of the data but also to generate diverse samples that conform to the training data distribution.

Qiao et al. (2024) proposed a joint enhancement framework integrating attention mechanisms and GAN. Multi-channel EEG signals are converted into brain cognition atlases as input. Feature representation is enhanced via the convolutional block attention module, while the GAN generates adversarial samples to expand the dataset. Classification is ultimately performed by a convolutional RNN. The results demonstrate that after GAN-based DA, the classification accuracy increased by approximately 2.5%.

Gilakjani and Osman (2024) designed an EEG emotion recognition framework combining contrastive learning and GAN. First, deep representations of EEG signals are learned in a self-supervised manner through contrastive learning. Then, GANs are employed to synthesize emotion-category-related features to expand the training set. Finally, the features are fed into a GNN for emotion classification. On the DEAP dataset, adding a volume of synthetic samples equivalent to the real samples increased valence classification accuracy from 59.37 to 64.85%, and arousal classification accuracy from 62.10 to 66.40%. On the MAHNOB-HCI dataset, adding half the volume of synthetic samples improved valence classification accuracy from 66.03 to 66.98%, and arousal classification accuracy from 61.32 to 71.69%.

Tian et al. (2023) extracted DE features from 5 frequency bands of EEG signals and constructed two types of feature representations: temporal morphology data and spatial morphology data. They then used a dual-encoder to extract spatiotemporal features separately, concatenated the latent variables of both encoders, and fed them into a decoder to generate synthetic samples. The model achieved an optimal average classification accuracy of 97.21% on the SEED dataset when augmenting with 15,000 samples, representing a 5% improvement over the original dataset.

Zhang et al. (2023) proposed a self-supervised DA framework called GANSER, comprising an adversarial augmentation network (AAN) and a multi-factor training network (MTN). The AAN randomly masks segments of EEG signals via a masking transformation operation and integrates a GAN to synthesize high-quality EEG samples, using a masking probability threshold as an uncertainty control mechanism to ensure diversity. It also employs a spatial–temporal network to capture spatiotemporal features of EEG data. The MTN designs a multi-factor self-supervised loss function that leverages masking probability as prior knowledge to optimize feature extraction, enhancing classifier generalizability through simulated signals. Results on the DEAP dataset show that the GAN and masking transformation design improves model performance by approximately 1%.

Ari et al. (2022) proposed a DA method based on a wavelet activation function-based extreme learning machine wavelet autoencoder (ELM-W-AE). They used CWT to convert EEG signals into time-frequency spectrograms (Scalograms), and subsequently employed ELM-W-AE for DA. The core innovation lies in adopting wavelet functions as replacements for traditional activation functions, including Gaussian, groove gap waveguide (GGW), Mexican, Meyer, Morlet, and Shannon. The augmented samples were classified by a fine-tuned residual network-18 (ResNet-18) network, achieving a classification accuracy of 99.6% on the GAMEEMO dataset—an improvement of approximately 22% compared to non-augmented data. Among these, the GGW wavelet function performed optimally, achieving 99.6% accuracy.

Kalashami et al. (2022) extracted 344-dimensional EEG features and adopted a DA method based on a conditional wasserstein GAN to generate synthetic feature samples. Results demonstrated that adding 5,000 synthetic samples improved the DNN’s recognition accuracy for arousal and valence by 6.5 and 3.1%, respectively.

Pan and Zheng (2021) extracted power spectral density (PSD) features from EEG signals in the θ , α , β , and γ frequency bands and augmented the dataset using a PSD-GAN. The generator comprises three linear layers with two rectified linear unit (ReLU) activations and one hyperbolic tangent activation, while the discriminator consists of two linear layers with leaky ReLU and sigmoid activations. On the DEAP dataset’s binary classification tasks, their DA method improved within-subject recognition accuracy for valence and arousal by averages of 5.25 and 6.38%, respectively, with cross-subject improvements of 6.5 and 6.71%; for four-class classification, it achieved within-subject and cross-subject accuracy gains of 10.92 and 14.47%. Meanwhile, on the MAHNOB-HCI dataset, augmentation elevated frequency band correlation CNN accuracy from 62.06 to 70.34%, and frequency band separation CNN accuracy from 56.78 to 66.5%.

Luo et al. (2020) proposed three DA methods based on VAE and GAN: conditional wasserstein GAN (cWGAN), selective VAE (sVAE), and selective WGAN (sWGAN). For the SEED dataset, when using sWGAN for DA and DNN as the classifier, the DE features improved the average accuracy by 10.2%. On the DEAP dataset, adopting sWGAN for augmentation with SVM as the classifier yielded a 5.4% increase in average accuracy. The findings indicate that sWGAN achieves the highest accuracy, although it demands more computation time than cWGAN. Additionally, they found that the volume of generated data should be less than 10 times the size of the original training set to achieve optimal model performance.

Zhu et al. (2024) convert DE features from EEG signals into spatiotemporal-frequency representations as input. The VAE-GAN module generates synthetic EEG samples to double the training dataset. Classification is then performed by a depthwise over-parameterized convolutional and transformer network. Results demonstrate that VAE-GAN augmentation improved accuracy by 1.01% for arousal and 1.14% for valence on DEAP, and by 2.45% on SEED.

Wan et al. (2025) introduced an EEG DA method leveraging generative transformer and contrastive learning. The approach decomposes raw EEG signals into time-invariant and time-specific components via multi-task learning, while aligning cross-subject data into a shared representation space to ensure invariance. Evaluations on DEAP, SEED, and SEED-IV datasets demonstrated significant performance gains: Accuracy improved by 4.5–10.5% across datasets for models trained with augmented data, with cross-stimuli tests confirming generalization to unseen emotional contexts. Unlike GAN/VAE-based augmentation, this method uniquely employs spatiotemporal decomposition and subject-invariant alignment to enhance robustness.

Beyond reporting accuracy gains, studies on generative augmentation have also evaluated the quality of synthetic samples in several ways. The most common approach is indirect validation through downstream emotion recognition performance, including comparisons with and without generated data and tests of generalization under cross-subject or cross-stimuli settings (Gilakjani and Osman, 2024; Ari et al., 2022; Pan and Zheng, 2021; Zhu et al., 2024; Wan et al., 2025). Some studies further examine the similarity between real and generated data using visual or feature-space analyses, such as direct sample comparison, topographic maps, principal component analysis, or t-distributed stochastic neighbor embedding (Qiao et al., 2024; Zhang et al., 2023; Kalashami et al., 2022; Luo et al., 2020). More rigorous evaluations additionally incorporate distribution-based metrics and statistical tests, including Kullback–Leibler divergence, Jensen–Shannon divergence, Earth Mover’s distance, *t*-tests, and rank-sum tests (Tian et al., 2023). These results suggest that the quality of synthetic EEG should be assessed not only by downstream performance gains, but also by its similarity to real data and its statistical consistency.

Generative modeling approaches represent one of the most promising directions for EEG DA. Unlike traditional transformation-based techniques, generative models aim to learn the underlying data distribution and synthesize entirely new samples that resemble real EEG signals (Lei et al., 2024). The theoretical assumption behind these methods is that EEG signals are generated from an underlying latent distribution, and that a well-trained generative model can approximate this distribution and sample new instances from it while preserving the statistical and spatiotemporal characteristics of real EEG data (You et al., 2025). The reviewed studies consistently report noticeable improvements in classification performance, particularly when synthetic data are generated in moderate quantities. In addition, generative approaches can capture complex spatiotemporal dependencies within EEG signals, enabling the creation of more diverse training datasets (Qiao et al., 2024). However, these methods typically require substantial computational resources and careful training procedures to ensure stability and avoid issues such as mode collapse or poor sample quality (You et al., 2025). Furthermore, excessive generation of synthetic samples may introduce distributional bias, suggesting that the ratio between real and generated data must be carefully controlled (Tian et al., 2023; Luo et al., 2020).

#### 3.4.5 Sampling

Sampling refers to selecting a smaller subset from a larger dataset to estimate characteristics of the entire data (Siuly et al., 2015). Sampling techniques can effectively address class imbalance issues in datasets. Based on strategies for handling imbalanced data, sampling methods fall into two main categories: oversampling and subsampling (undersampling). Oversampling balances class distribution by increasing the number of minority class samples, while subsampling achieves balance by reducing majority class samples (Kummer et al., 2022).

Synthetic minority over-sampling technique (SMOTE) is an oversampling method. For each minority class sample, the SMOTE algorithm calculates the distance to its nearest neighbors, then generates new samples by interpolating between the original sample and its neighbors (Kummer et al., 2022). However, SMOTE has limitations. By generating synthetic samples through interpolation within minority classes, it may cause overlap between newly created minority samples and majority samples, thereby blurring decision boundaries and degrading classifier performance (Udu et al., 2025). Borderline-SMOTE improves upon SMOTE by focusing specifically on minority samples near the boundary between minority and majority classes. By oversampling only these boundary samples, borderline-SMOTE generates synthetic data that more effectively clarifies decision boundaries, enhancing the classifier’s ability to distinguish between minority and majority classes (Chen et al., 2021).

Chen et al. (2021) addressed the issue of imbalanced sample distribution across emotion classes in the DEAP dataset. They first extracted frequency-domain features from EEG signals using the STFT. Then, the borderline-SMOTE algorithm was applied to oversample minority-class samples near the classification boundaries, generating synthetic samples to balance the data distribution. Finally, a one-dimensional CNN was used to perform three-class classification tasks on the valence and arousal dimensions. This approach achieved average accuracies of 97.47% for valence and 97.76% for arousal, representing an improvement of approximately 5% compared to the non-augmented baseline.

Singh K. et al. (2023) proposed a subject-wise DA method based on a balancing factor. In this study, the DEAP dataset was used, where EEG signals were first segmented. For each subject, a balancing factor for the distribution of the four emotion classes was calculated. Synthetic samples, consistent with the distribution of the original EEG signals, were generated for the minority classes using SMOTE, a linear interpolation method based on k-nearest neighbors, to balance the sample sizes across the four emotion classes. Finally, emotion classification was performed using a one-dimensional CNN and bidirectional LSTM hybrid model. After DA, the model’s precision, recall, F1-score, accuracy, and specificity improved by an average of 6.02, 9.44, 7.98, 6.01, and 2.66%, respectively.

The reviewed studies demonstrate that sampling strategies can significantly improve evaluation metrics such as recall and F1-score, particularly in multi-class emotion recognition tasks. The theoretical assumption underlying these methods is that samples belonging to the minority class occupy a continuous local region in the feature space, and that generating new samples through interpolation among neighboring minority instances can approximate the true data distribution while balancing class representation (Chawla et al., 2002). Nevertheless, interpolation-based methods may produce synthetic samples that overlap with majority-class regions in the feature space, potentially blurring decision boundaries (Li Y. et al., 2025). Therefore, improved variants such as borderline-SMOTE attempt to focus on samples near classification boundaries to mitigate this issue (Carvalho et al., 2025). Overall, sampling strategies are most effective when class imbalance is severe, but are less suitable as a standalone augmentation technique for enhancing signal diversity.

#### 3.4.6 EMD

EMD is a signal processing technique designed to decompose EEG signals into multiple intrinsic mode functions (IMFs) and a residual component (Ge et al., 2018). Each IMF represents distinct frequency components within the signal, while the residual contains low-frequency elements or trend terms. By applying weighting, reorganizing, or introducing random perturbations to these IMFs, new signals can be synthesized.

Singh U. et al. (2023) employed EMD to decompose raw EEG signals into multiple IMFs. By calculating the dynamic time warping distance, Pearson correlation coefficient, and Spearman correlation coefficient, they selected the IMF component with the highest correlation to the original signal as synthetic samples, thereby increasing the total sample size by 50%. Experiments validated on the DEAP dataset demonstrated that this approach effectively enhanced the performance of emotion classification models, achieving accuracies of 92.5% for valence and 81.25% for arousal in binary classification tasks.

Aldawsari et al. (2023) proposed a DA method based on graph EMD. This approach models multi-channel EEG signals as graph structures, decomposes the original signals to generate graph intrinsic mode functions (graph-IMFs), and synthesizes new samples by cross-sample recombination of graph-IMFs from the same emotion category. On the DEAP, MAHNOB-HCI, and SEED datasets, doubling the data volume increased classification accuracy by 4.8, 3.9, and 3.5%, respectively.

EMD-based augmentation methods are particularly suitable for non-stationary signals such as EEG, as they preserve the multi-scale frequency characteristics inherent in neural activity (Raveendran et al., 2024). This method assumes that EEG signals can be decomposed into IMFs representing different frequency components, and that recombining these components can generate new signals while preserving essential signal characteristics (Ge et al., 2018). The reviewed studies indicate that EMD-based augmentation can moderately improve classification performance while maintaining physiologically plausible signal structures. However, the number of studies adopting this method remains relatively limited, and the effectiveness of different IMF recombination strategies has not yet been systematically evaluated. Additionally, the computational cost of signal decomposition may increase preprocessing complexity, which may limit its scalability in large datasets (Raveendran et al., 2024).

#### 3.4.7 Other

Wang et al. (2024a) conducted a study on cognitive workload classification during simulated flights using various DA methods, including sliding window, Fourier Transform Surrogates (FTS), mixup, and combinations of these techniques. Experimental results demonstrated that FTS outperformed sliding window and mixup in cross-clip scenarios, achieving an accuracy of 90.36%, while mixup was better suited for cross-session scenarios. When combining all three DA methods, the model achieved optimal classification results in both scenarios. The cross-clip classification accuracy reached 90.63% (an 8.46% improvement compared to no enhancement), and the cross-session classification accuracy reached 86.73% (a 3.47% improvement compared to no enhancement).

Zhou R. et al. (2025) designed EEG-mixup, a specialized DA strategy for EEG signals. Unlike standard mixup, which ignores EEG’s non-stationary nature, EEG-mixup strictly blends segments from the same subject and trial to maintain valid data distributions. For labeled data, it generates new samples by mixing both EEG signals and their emotion labels. For unlabeled data, it augments EEG signals only. This approach significantly expanded training data while preserving EEG characteristics, contributing to the model’s top performance (e.g., 91.35% accuracy on SEED with limited labels) and outperforming standard mixup by 3.57%.

Jin et al. (2024) adopted a DA method based on random temporal disruption. This approach first constructs an energy tensor from relative energy matrices. Leveraging the characteristic that the temporal axis order in the energy tensor is uncorrelated with mental workload, the sequence of time windows in the original tensor is randomly permuted. This method expanded the sample size by 30 times. The augmented data was then fed into a classifier designed using deep transfer learning and depthwise separable CNNs. Results on their self-collected dataset showed that DA improved the model’s accuracy from 83.33 to 97.22%, an increase of 13.89%.

Bagherzadeh et al. (2024) first converted single-channel EEG signals into two-dimensional time-frequency images using the synchrosqueezing wavelet transform. They then augmented the training set by integrating time-frequency maps from all channels of all subjects except the target subject. This DA method significantly increased the sample size while avoiding the destruction of inherent structures in EEG time-frequency maps caused by traditional geometric image transformations. Finally, the augmented two-dimensional time-frequency images were fed into a ResNet-18. The experiment achieved average accuracies of 76.66, 78.12, 81.25, and 75.00% for recognizing sad and happy emotions in the SEED-IV, SEED-V, SEED-GER, and SEED-FRA databases, respectively.

Kan et al. (2022) were inspired by genetics and proposed a self-supervised group meiosis contrastive (SGMC) learning framework based on stimuli-consistent EEG signals. They designed a meiosis DA method, treating multi-subject EEG signals as “chromosomes” by leveraging the stimulus consistency within EEG sample groups. Augmented groups were generated through pairing, cross-exchanging, and separation. The model extracts cross-subject group-level feature representations via a group projector and employs contrastive learning to maximize the similarity between original and augmented groups under identical stimuli. Experimental results demonstrate that without DA, accuracy on the DEAP and SEED datasets decreased by 3 and 2%, respectively, confirming the critical role of meiosis DA in enhancing emotion recognition within SGMC.

Kang et al. (2022) proposed an ICA and evolutionary algorithm-based DA framework (ICA-E). The method first decomposes EEG signals via ICA, extracts independent components (ICs) for each emotion category, and removes noise components. Next, in the component space, mutual information serves as the fitness function to screen key ICs, while new ICs are generated through time-window segmented crossover recombination. Finally, in the sensor space, mutation is achieved via linear combinations of rotated reconstructed signals, and augmented data is generated by combining mutation with crossover recombination. They input the augmented spectrograms into an ensemble model combining CNN and LSTM. Experimental results demonstrate that when augmenting 200 trials per class, the model’s recognition accuracy on the DEAP dataset reached 84.92%, outperforming other traditional DA methods.

Zhang D. et al. (2024) proposed a wavelet transform-based DA method. Their approach applied CWT with Morlet wavelets across 10 scales, decomposing EEG signals into multi-resolution representations. These were recombined into new 2D matrices, significantly expanding the original dataset while preserving spatiotemporal features. For the DEAP emotion recognition dataset, this augmentation achieved 92% accuracy, outperforming feature-fusion methods by 4%.

Chen et al. (2025) proposed an uncertainty-guided graph augmentation (UGA) that decomposes EEG graphs into domain-specific and invariant components. It then probabilistically transforms both domain-specific features and connectivity by sampling new statistical characteristics based on batch-wise uncertainties. This generates augmented graphs mimicking cross-subject distribution shifts while preserving emotional semantics. On SEED, SEED-IV, and SEED-V datasets, the UGA-enhanced network achieved cross-subject accuracies of 93.11, 77.16, and 77.12%, respectively.

The remaining DA techniques include various emerging strategies such as FTS, meiosis and ICA-E. These approaches typically exploit specific properties of EEG signals, such as temporal invariance or frequency-domain characteristics, to construct new training samples. Compared with traditional DA methods, these strategies often aim to improve representation learning rather than merely expanding the dataset size. Although several studies report promising performance improvements, the diversity of methodologies and experimental settings makes systematic comparison difficult. Consequently, further research is required to evaluate the generalizability of these emerging techniques across different datasets, tasks, and model architectures.

## 4 Discussion

### 4.1 Findings

#### 4.1.1 Input formulation and classification models

**Figure 4:**
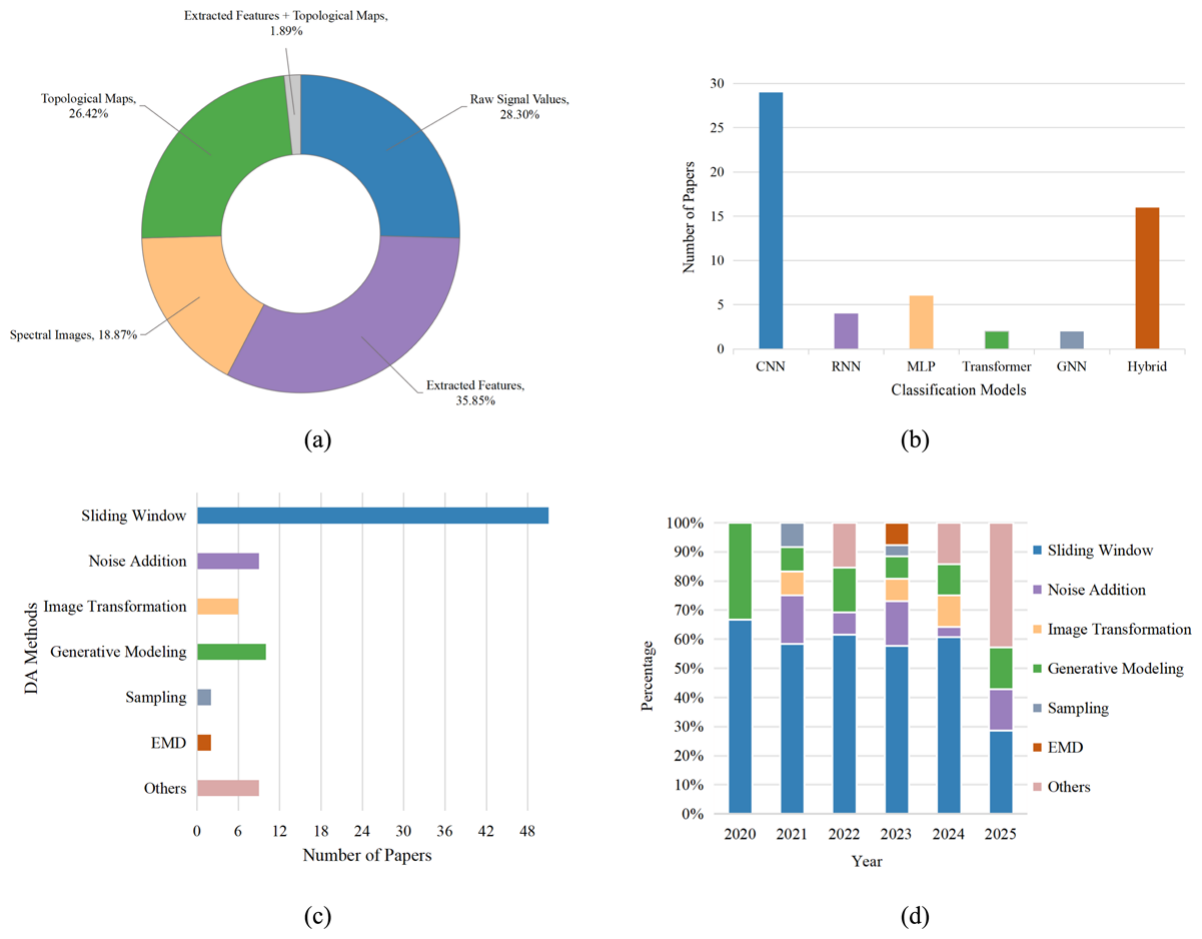
Preliminary analyses. **(a)** The frequency of usage for each input formulation, **(b)** the number of times different deep learning-based classification models are used, **(c)** the number of uses for DA methods, and **(d)** usage frequency of different DA methods from 2020 to 2025.

In this study, researchers often use extracted features and raw signal values as inputs to deep learning models. Using extracted features highlights the advantages of feature matrices in preserving frequency-domain characteristics and maintaining compatibility with traditional classifiers, while also demonstrating that end-to-end time-series inputs more effectively capture temporal dynamics. Figure 4a illustrates the frequency of usage for each input formulation. Figure 4b displays the number of times different deep learning-based classification models are used. CNN is the most popular deep learning-based classification model. This is primarily because CNNs excel at processing spatially and temporally localized features, making them particularly well-suited for handling large-scale, complex EEG data. Their strengths in pattern recognition enable effective extraction of discriminative features from EEG signals, thereby improving classification accuracy. Notably, hybrid models are also widely adopted, emerging as the second most prevalent model choice after CNNs. Such models achieve multi-level feature fusion by seamlessly integrating the complementary strengths of CNNs, RNNs, and other architectures.

#### 4.1.2 DA

DA serves as a key technique for mitigating the small-sample constraints in deep learning, with various strategies widely implemented (Wang et al., 2025a; He et al., 2021; Lashgari et al., 2020). In this review, we categorize DA techniques for EEG-based emotion recognition and cognitive workload decoding under deep learning into seven classes: sliding window, noise addition, image transformation, generative modeling, sampling, EMD, and others. Figure 4c illustrates the number of uses for these DA methods. Sliding windows represent the most commonly employed DA method. Most studies utilize non-overlapping sliding windows for data segmentation, adopting different window sizes, with 1 s being the most prevalent. For overlapping sliding windows, some researchers have experimented with varying window sizes or step lengths, though consensus on optimal values is lacking. Noise addition, as a critical strategy for enhancing model robustness, has demonstrated significant effectiveness across three data formats: raw signals, extracted features, and visual representations of EEG signals (e.g., spectral images, topological maps). Its efficacy, however, depends heavily on fine-tuning the noise parameters. In image-based EEG representations, single image transformation techniques may sometimes be ineffective, or even harm classification performance. Consequently, most studies employ combined strategies to improve classification outcomes. Generative models such as GANs and AEs, while showing promise in augmenting EEG data, have not been proven superior to simpler methods like noise addition in any study. Sampling techniques are mainly used to address class imbalance, playing a particularly critical role in emotion recognition tasks. EMD enhances data by decomposing signals into IMFs, achieving both DA and noise separation. This approach exhibits advantages in preserving physiological features and expanding sample size.

Furthermore, Figure 4d illustrates the temporal evolution of different DA methods from 2020 to 2025. Sliding window remains the most dominant technique across all years. Noise addition maintains relatively stable usage due to its simplicity and effectiveness in improving model robustness, while image transformation methods are increasingly adopted in studies using image-based EEG representations. In recent years, more advanced generative approaches have begun to attract attention, including transformer-based generative models for synthesizing realistic EEG samples. In addition, several emerging augmentation strategies have been introduced in recent studies, such as random temporal disruption, cross-subject channel sampling, and statistical transformation, reflecting growing efforts to design more sophisticated and task-specific augmentation mechanisms. Overall, these results suggest a gradual shift from traditional segmentation and perturbation-based methods toward more diverse and innovative DA strategies.

In addition, the suitability of DA methods appears to depend strongly on the input representation of EEG data. For raw signal inputs, signal-level strategies such as sliding window, noise addition, EMD, and temporal recombination are generally more appropriate because they preserve temporal dynamics and can be directly integrated into end-to-end models. For extracted features, feature-space perturbation, oversampling methods such as SMOTE, and feature-level generative models are often more suitable, as they operate on compact discriminative representations and are effective for class balancing. For spectral images, image-based DA methods, including geometric transformations, brightness or color perturbation, and image-domain generative models, are more naturally compatible with CNN-based pipelines. However, excessive transformations may distort the time-frequency semantics of EEG-derived images. For topological maps, augmentation should preserve the spatial organization of electrodes and inter-channel relationships; therefore, topology-aware transformations and graph-based augmentation strategies are generally more appropriate than arbitrary image manipulations. Overall, the reviewed studies suggest that no single DA method is universally optimal across all EEG representations, and representation-aware selection is essential for achieving robust and physiologically meaningful augmentation.

#### 4.1.3 Evaluation protocols for DA

**Figure 5:**
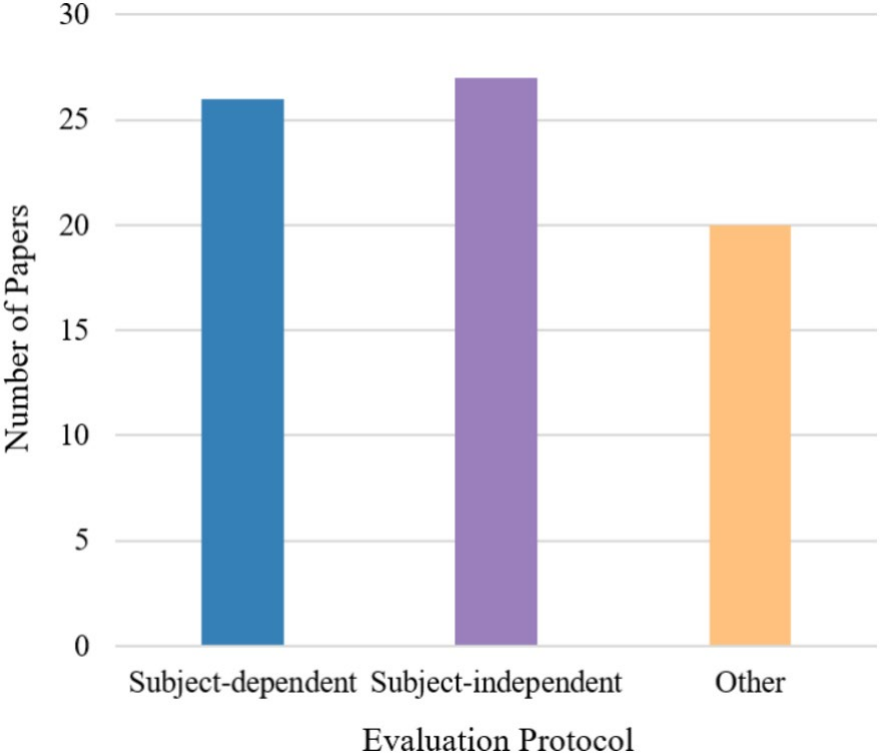
Number of papers using each evaluation protocol.

We further analyzed the evaluation protocols under which DA methods were assessed in the 59 reviewed studies. As shown in Figure 5, 26 studies used subject-dependent settings, 27 employed subject-independent (cross-subject) protocols, and 20 adopted other strategies, such as cross-task, cross-database, cross-session, or non-standard partitions. Some studies reported results under multiple protocols.

The nearly balanced use of subject-dependent and subject-independent evaluations suggests increasing attention to cross-subject generalization in EEG-based emotion recognition and cognitive workload decoding. However, a considerable proportion of studies still relied on subject-dependent or non-standard settings, which may overestimate the practical effectiveness of DA methods because they do not adequately capture inter-subject variability. In addition, the diversity of evaluation protocols makes direct comparison across studies difficult. A DA method that performs well under subject-dependent settings may not remain effective in subject-independent scenarios. Therefore, future work should emphasize rigorous subject-independent evaluation and clearly report experimental protocols to support fair comparison. Establishing standardized cross-subject benchmarks would further facilitate systematic assessment of DA methods and their true generalization ability.

### 4.2 Problems

#### 4.2.1 Insufficient specialized research and lack of comparability

Although the value of DA in EEG-based deep learning has gained increasing recognition, specialized research focusing specifically on emotion recognition and cognitive workload decoding is still limited. Existing studies mostly focus on tasks like MI and epilepsy detection (He et al., 2021; Lashgari et al., 2020). Systematic research comparing the performance of different DA methods within the same task is scarce, making it difficult to assess their applicability. Additionally, the comparability of research findings is constrained by variations in classification models, input formats, and hyperparameter settings, making it difficult to directly measure the effectiveness of the DA methods themselves.

#### 4.2.2 Lack of theoretical guidance for DA

Firstly, there is no solid theoretical basis or quantitative guidelines for determining the optimal number of new samples to generate. Insufficient augmentation fails to effectively mitigate overfitting, while excessive augmentation risks introducing noise or disrupting inherent data patterns (e.g., pattern collapse), leading to performance degradation (Luo et al., 2020; Kang et al., 2022). The current selection of augmentation volume primarily relies on empirical tuning. Secondly, key DA parameters (e.g., sliding window step size, noise intensity σ , GAN latent space dimension, number of neighbors k in SMOTE) significantly impact effectiveness, but their optimal settings are typically highly task- and model-dependent, making them difficult to generalize.

#### 4.2.3 Separation between the model and DA

Current research on DA in EEG-based deep learning fails to systematically incorporate the specific characteristics of model architectures as the core basis for designing and selecting augmentation strategies. The two aspects—model design and augmentation strategy—exist in a relatively independent and disjointed state. Applying augmentation strategies generically in this manner may render them ineffective or even detrimental for certain models. This hinders the goal of maximizing model potential through tailored DA.

### 4.3 Future trend

#### 4.3.1 Establishing standardized evaluation frameworks

To address the current weaknesses in the research foundation and the poor comparability among methods in EEG-based emotion recognition and cognitive workload decoding, a primary future direction is to construct standardized evaluation frameworks (Yang et al., 2024; Ma et al., 2025; Islam et al., 2024). These frameworks should define publicly accessible benchmark datasets, establish unified baseline models, input formulations, core evaluation metrics, and strict assessment protocols (Sun Q. et al., 2025). Based on this foundation, large-scale comparative studies should be conducted to quantify the performance differences among various DA techniques under identical tasks, models, and evaluation criteria. This will clarify the optimal DA strategies and parameter ranges for different scenarios, providing a reproducible and comparable basis for subsequent research.

#### 4.3.2 Deepening quantitative research on DA

To resolve the core issue of lacking theoretical guidance on sample quantity and key parameter settings in DA strategies, researchers should intensively explore the relationship between the number of generated samples and factors such as model performance, model complexity, original dataset size, and task difficulty. The goal is to establish theoretical or empirical guidelines (e.g., augmentation ratio thresholds) for selecting the optimal augmentation volume, preventing under- or over-augmentation (More and Bradbury, 2025). Simultaneously, quantitative relationships between key augmentation parameters (e.g., window parameters, noise levels, generative model parameters) and enhancement effectiveness should be studied to provide a more scientific basis for parameter configuration (Szczakowska et al., 2023; Jiang et al., 2023; Ari et al., 2022). Future research could further incorporate computational mathematics methods to enhance the precision of DA, such as leveraging rigorous error analysis in quaternion operations (Zhang et al., 2025; Zhang F. et al., 2024) and structure-preserving matrix decomposition techniques (Wei et al., 2024; Sun J. et al., 2025), to ensure numerical stability and efficiency in generative DA models for EEG signal processing.

#### 4.3.3 Developing model-collaborative DA

Future research could explore multi-level, procedural augmentation frameworks that organically integrate techniques across different hierarchies (e.g., signal-level, feature-level, image-level) (Yin et al., 2024; Mane and Shinde, 2023). Researchers need to customize the selection or combination of the most effective DA strategies based on the intrinsic characteristics and learning states of deep models. Furthermore, techniques like reinforcement learning could be leveraged to automatically search for and optimize key augmentation parameters (e.g., noise intensity σ , sliding step size, SMOTE’s k -value) based on a model’s real-time performance feedback on validation sets (Wang et al., 2025b). This enables dynamic adaptation of augmentation strategies.

## 5 Conclusion

This paper systematically reviews the application of EEG DA techniques in emotion recognition and cognitive workload decoding within deep learning frameworks over the past 5 years. Based on the analysis of 59 core publications, the study elaborates on the experimental paradigms of four major public datasets: DEAP, SEED-series, DREAMER, and MAHNOB-HCI. It also categorizes four primary EEG input formulations: raw signal values, extracted features, spectral images, and topological maps, and classifies six mainstream deep learning models: CNNs, RNNs, MLPs, GNNs, transformers, and hybrid models. The core contribution lies in an in-depth analysis of seven categories of DA methods: sliding window, noise addition, image transformation, generative modeling, sampling, EMD, and others. Furthermore, the paper discusses key findings, such as the dominance of CNNs and the prevalent use of sliding windows, along with current challenges and critical future research directions.

In summary, this review provides valuable guidance for researchers to understand the mechanisms of EEG DA in this domain and to select appropriate methods for enhancing model generalizability, while laying the methodological groundwork for addressing cross-subject and small-sample challenges in emotion and cognitive workload decoding.
